# LGRs in Skeletal Tissues: An Emerging Role for Wnt‐Associated Adult Stem Cell Markers in Bone

**DOI:** 10.1002/jbm4.10380

**Published:** 2020-07-03

**Authors:** Laura Doherty, Archana Sanjay

**Affiliations:** ^1^ Department of Orthopaedic Surgery UConn Health Farmington CT USA

**Keywords:** STROMAL/STEM CELLS, WNT/β‐CATENIN, MOLECULAR PATHWAYS–REMODELING, OSTEOBLASTS, INJURY/FRACTURE HEALING

## Abstract

Leucine‐rich repeat‐containing G protein‐coupled receptors (LGRs) are adult stem cell markers that have been described across various stem cell niches, and expression of LGRs and their corresponding ligands (R‐spondins) has now been reported in multiple bone‐specific cell types. The skeleton harbors elusive somatic stem cell populations that are exceedingly compartment‐specific and under tight regulation from various signaling pathways. Skeletal progenitors give rise to multiple tissues during development and during regenerative processes of bone, requiring postnatal endochondral and intramembranous ossification. The relevance of LGRs and the LGR/R‐spondin ligand interaction in bone and tooth biology is becoming increasingly appreciated. LGRs may define specific stem cell and progenitor populations and their behavior during both development and regeneration, and their role as Wnt‐associated receptors with specific ligands poses these proteins as unique therapeutic targets via potential R‐spondin agonism. This review seeks to outline the current literature on LGRs in the context of bone and its associated tissues, and points to key future directions for studying the functional role of LGRs and ligands in skeletal biology. © 2020 The Authors. *JBMR Plus* published by Wiley Periodicals, Inc. on behalf of American Society for Bone and Mineral Research.

## Introduction

Leucine‐rich repeat‐containing G protein‐coupled receptors (LGRs) are a family of adult stem cell surface proteins that have been studied in organs with well‐defined stem cell niches, including the hair follicle and intestine.^(^ ^1^, ^2^ ^)^ The LGR family members (LGR4/5/6) mark distinct cells with specialized functions during homeostasis and stress responses, mainly in Wnt‐driven progenitor compartments,^(^ ^3^, ^4^ ^)^ and play a critical role in defining progenitor and stem cell behavior.^(^ ^2^, ^5^ ^)^

Skeletal stem cells are required for bone formation during development, for proper differentiation of functional osteoblasts during homeostatic bone remodeling, and for providing osteochondral progenitors during postnatal regenerative processes. The complex anatomy of bone and bone‐associated tissues that participate in these processes requires that skeletal stem cells are highly compartmentalized. As evident from studies in Wnt‐dependent cell populations in intestine, hair follicle, and lung, many organs maintain distinct adult stem cell populations with varying functions. These separate populations are especially delineated and appreciated following tissue injury, and, in many cases, are marked by specific LGRs.^(^ ^1^, ^6^, ^7^ ^)^ Recent advances in the field of skeletal biology and regeneration have identified possible candidate markers for distinct stem cell populations within bone,^(^ ^8^ ^)^ including within the context of fracture healing.^(^ ^9^, ^10^, ^11^, ^12^, ^13^ ^)^ Expression of various LGRs and R‐spondins has now been described in skeletal cell types, including osteoblasts and osteoclasts, as well as their progenitor populations.^(^ ^14^ ^)^

**Fig 1:**
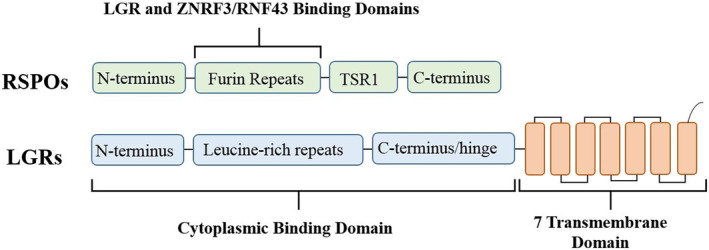
Structural schematic of leucine‐rich repeat‐containing G protein‐coupled receptors (LGRs) and their ligands, R‐Spondins (RSPOs). All LGR family members contain leucine‐rich repeats fused to a 7 transmembrane domain at the C‐terminus hinge region. RSPOs contain a thrombospondin type I repeat domain (TSR1), and two furin repeats that compose the binding domain for LGRs.

Structurally, LGRs are G protein‐coupled receptors belonging to a class A rhodopsin‐like family.^(^ ^15^ ^)^ They are a conserved group of 7‐transmembrane proteins with characteristic leucine‐rich repeats in the N‐terminal extracellular domain that can mediate ligand interaction.^(^ ^16^, ^17^, ^18^ ^)^ A family of secreted proteins called R‐Spondins (RSPOs) have been identified as ligands for LGRs,^(^ ^19^, ^20^, ^21^, ^22^, ^23^ ^)^ where all mammalian R‐spondins (RSPO1–4) share a similar protein structure with two furin‐like repeat domains that act as the binding domain for the receptors (Fig. 1).^(^ ^22^, ^24^ ^)^ Although each of the LGRs have a similar and highly conserved structure, LGR4 and LGR5 feature 17 leucine‐rich repeats, whereas LGR6 contains 13 repeats.^(^ ^16^ ^)^ Despite the fact that LGRs constitute a subfamily within the G protein‐coupled receptor (GPCR) superfamily,^(^ ^25^ ^)^ LGR/RSPO binding does not result in canonical activation of these receptors via signal transduction through either cAMP or the IP3/DAG pathway.^(^ ^19^, ^20^, ^21^ ^)^ Therefore, LGRs are unique GPCRs with specialized functions, one of which has been recently elucidated in the Wnt signaling pathway.

**Fig 2:**
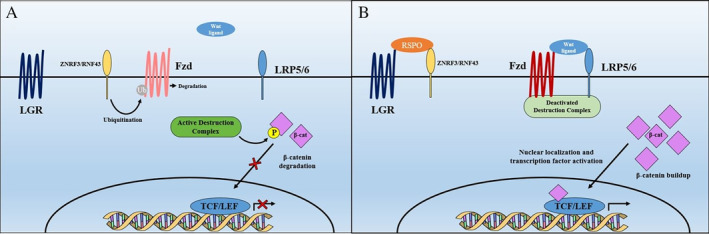
Current understanding of leucine‐rich repeat‐containing G protein‐coupled receptors (LGRs) as auxiliary receptors in the Wnt pathway. (*A*) In the absence of R‐spondins, the ubiquitination complex ZNRF3/RNF43 targets Fzd for degradation, removing receptor binding sites for Wnt ligands. The destruction complex is free to phosphorylate β‐catenin, preventing β‐catenin entry into the nucleus and leading to attenuated Wnt signaling. (*B*) R‐spondins bind to LGRs and sequester the ubiquitination complex ZNRF3/RNF43. This results in longer cell surface residence of Fzd, and in the presence of Wnt ligands, the destruction complex is deactivated. Free from phosphorylation and degradation, β‐catenin can enter the nucleus to bind TCF/LEF, driving the transcription of Wnt target genes and enhancing Wnt signaling.

Within the Wnt/β‐catenin pathway, the transmembrane ubiquitin ligases ZNRF3 and RNF43 act to degrade Frizzled (Fzd) receptors, the key receptors for Wnt family ligands (Fig. 2*A*). LGRs can act as auxiliary receptors in this pathway, where binding of the various soluble R‐spondin ligands sequesters the ubiquitination complex ZNRF/RNF43, prolonging the cell surface residence of Fzd.^(^ ^26^, ^27^ ^)^ In the canonical pathway, this lengthened period of Fzd exposure at the cell surface poses more opportunities for the binding of Wnt ligands, resulting in increased accumulation of stabilized β‐catenin (Fig. 2*B*).^(^ ^28^ ^)^ The binding of nuclear β‐catenin to the transcription factors TCF/LEF leads to transcription of Wnt target genes, conferring the downstream effects of Wnt/β‐catenin signaling.^(^ ^28^, ^29^ ^)^

The canonical Wnt pathway is an important mediator of bone throughout the processes of development and homeostasis, as well as skeletal regeneration.^(^ ^30^, ^31^, ^32^ ^)^ In the context of bone, Wnt‐mediated target genes are numerous and include the essential transcription factors for osteoblast differentiation, including osterix (*Sp7*) and *Runx2*, the osteoblast‐specific hormone osteocalcin (*Ocn*), and *Rankl* and osteoprotegerin (*Opg*), both of which are involved in the formation of multinucleated osteoclasts.^(^ ^33^, ^34^, ^35^, ^36^, ^37^, ^38^ ^)^ Interestingly, LGRs themselves can constitute Wnt target genes, suggesting that Wnt activation is directly related to expression levels of these stem cell markers.^(^ ^2^ ^)^

**Fig 3:**
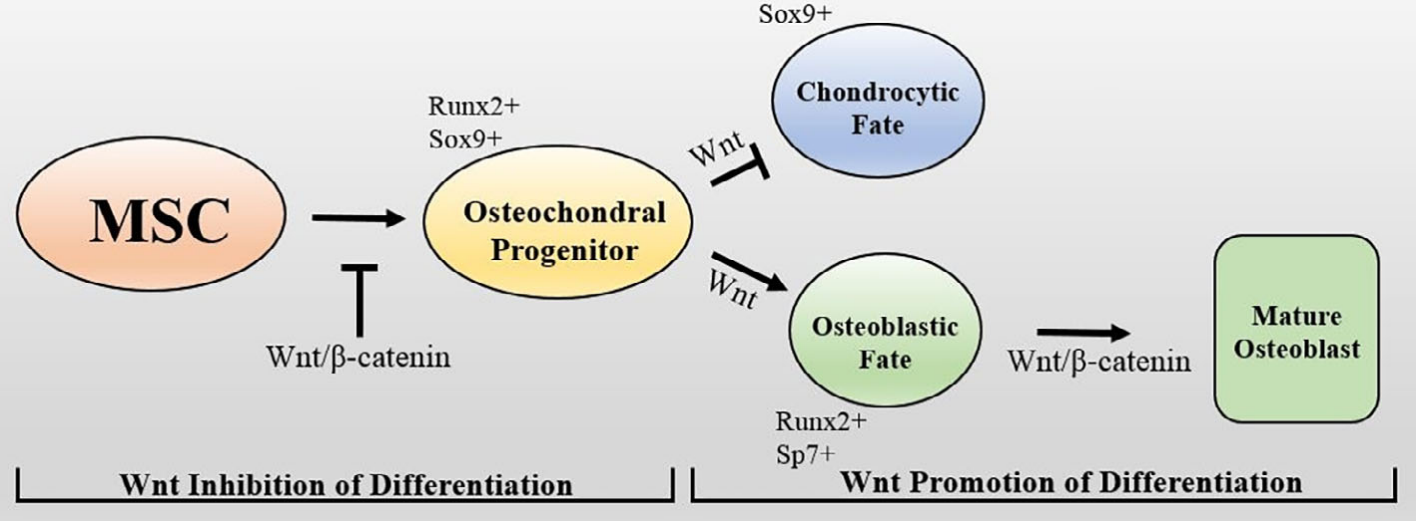
Wnt mediates osteogenic differentiation of mesenchymal stem cells (MSCs). Wnt/β‐catenin signaling inhibits skeletal progenitor specification. However, once cells have become committed osteochondral progenitors, Wnt signaling acts to potentiate differentiation toward the osteoblastic lineage.

Canonical Wnt signaling can either promote or inhibit osteogenic differentiation depending on the differentiation status or maturity of the cell. During bone formation, Wnt/β‐catenin activation keeps mesenchymal stem cells in a progenitor‐like state and prevents specification and commitment; conversely, in more committed cells, Wnt signaling further potentiates osteogenic differentiation of progenitors into mature osteoblasts (Fig. 3).^(^ ^39^, ^40^ ^)^ In the context of skeletal regeneration, activation of canonical Wnt signaling in the early phases of fracture healing has been shown to inhibit repair, whereas postfracture induction of Wnt activity actually enhances bone healing.^(^ ^41^, ^42^ ^)^ Targeting the Wnt pathway in bone using various Wnt ligands and inhibitors has therefore become a particularly important area of skeletal biology‐related research.^(^ ^43^ ^)^

Although some recent studies do demonstrate a modulation of Wnt signaling by LGRs, there is an important secondary aspect of LGRs as adult stem cell markers with no currently defined mechanism. This review briefly outlines LGR family member expression in bone, and how modulation of LGRs has been shown to affect skeletal and dental tissues (Table 1, Table 2).

**Table 1:** Summary of LGR Distribution and Expression in Bone, Teeth, and Their Associated Cells and Tissues

LGR	Cell/tissue	Source	Major findings	Reference
LGR4	Ameloblasts	Mouse	Expressed in ameloblasts of adult mouse incisors	Van Schoore et al.^(^ ^59^ ^)^
LGR4	Chondrocytes	Mouse	Deletion of LGR4 resulted in little change in chondrocytic marker genes, including *Col2a1*, *Ihh*, *Sox9*, and *Col10a1*, and has little effect on chondrocyte maturation in vivo at E14.5; expressed in cartilage of embryonic mice and in hyaline cartilage, elastic cartilage, and fibrocartilage of adult mice	Luo et al.^(^ ^48^ ^)^; Van Schoore et al.^(^ ^59^ ^)^
LGR4	Dental epithelium	Mouse	Required for sequential molar development controlled by Wnt signaling	Yamakami et al.^(^ ^79^ ^)^
LGR4	Developing Incisors	Mouse	Expressed in labial aspect of cervical loop at E18.5	Kawasaki et al.^(^ ^78^ ^)^
LGR4	Developing molars	Mouse	Strong expression in epithelium of dental lamina and bud stages, and later weakly expressed in odontoblasts beneath developing cusps	Kawasaki et al.^(^ ^78^ ^)^
LGR4	Developing limb	Mouse	E14.5 mesenchyme	Szenker‐Ravi et al.^(^ ^49^ ^)^
LGR4	Meckel's cartilage	Zebrafish	Present during zebrafish cranial development	Hirose et al.^(^ ^54^ ^)^
LGR4	Odontoblasts	Mouse	Expressed in odontoblasts of adult mouse incisors	Van Schoore et al.^(^ ^59^ ^)^
LGR4	Periosteum	Mouse	Expressed in adult periosteum	Van Schoore et al.^(^ ^59^ ^)^
LGR5	Developing Incisors	Mouse	Expressed in incisor stem cell niche and labial cervical loop at E18	Kawasaki et al.^(^ ^78^ ^)^; Suomalainen and Thesleff^(^ ^84^ ^)^
LGR5	Developing molars	Mouse	Expressed in mesenchyme buccal to bud tooth epithelium, with weak expression in collar of tooth epithelium during bud and cap stages	Kawasaki et al.^(^ ^78^ ^)^
LGR5	Developing limb	Mouse	E14.5 mesenchyme	Szenker‐Ravi et al.^(^ ^49^ ^)^
LGR5	Osteoblasts	Rat	Expression colocalizes with Runx2+ osteoblasts in alveolar bone during orthodontic murine tooth movement	Hosomichi et al.^(^ ^90^ ^)^
LGR5	PDL epithelial stem cells	Human	LGR5+ PDL cells coexpress markers of pluripotency	Athanassiou‐Papaefthymiou et al.^(^ ^91^ ^)^
LGR6	Blastemal mesenchyme	Mouse	LGR6+ mesenchymal cells from nailbed differentiate into osteoblasts during murine digit tip regeneration, and a subset of LGR6‐KO mice exhibit impaired blastema regeneration	Lehoczky et al.^(^ ^7^ ^)^
LGR6	Developing Incisors	Mouse	Expressed in mesenchyme adjacent to ameloblasts at E18.5	Kawasaki et al.^(^ ^78^ ^)^
LGR6	Developing molars	Mouse	Weak expression in epithelium during bud and cap stages, with later expression in dental mesenchyme. Strong expression in ameloblasts and odontoblasts	Kawasaki et al.^(^ ^78^ ^)^
LGR6	Developing limb	Mouse	Colocalizes with Wnt3 in the apical ectodermal ridge at E14.5	Szenker‐Ravi et al.^(^ ^49^ ^)^

**Table 2:** Summary of in vitro Studies on LGR Expression and Function in Skeletal Cells and Tissues

LGR	Cell/tissue	Source	Major findings	Reference
LGR4	2–14 cells (immature PDL cell line)	Human	Increased osteogenic differentiation in vitro under RSPO2 treatment	Arima et al.^(^ ^89^ ^)^
LGR4	Adipose‐derived stem cells	Human	Positively affects osteogenic differentiation via ERK/FGF signaling	Zhang et al.^(^ ^70^ ^)^
LGR4	Bone marrow‐derived mesenchymal stem cells	Mouse	Reduces in vitro osteogenic differentiation, upregulates proliferation	Luo et al.^(^ ^48^ ^)^; Sun et al., ^(^ ^62^ ^)^
LGR4	Calvarial osteoblasts	Mouse	Negatively regulates bone formation and kinetics via cAMP‐PKA‐Atf4 signaling	Luo et al.^(^ ^48^ ^)^; Pawaputanon na Mahasarakham et al.^(^ ^71^ ^)^
LGR4	MC3T3‐E1 cells (preosteoblast cell line)	Mouse	Promotes osteogenesis via RSPO2 binding; becomes upregulated upon BMP2 exposure	Luo et al.^(^ ^48^ ^)^; Zhu et al.^(^ ^68^ ^)^; Pawaputanon na Mahasarakham et al.^(^ ^71^ ^)^; Zhou et al.^(^ ^67^ ^)^
LGR4	Preosteoclasts, osteoclasts	Mouse	Soluble extracellular domain binds RANKL and inhibits hyperactivation of osteoclasts	Luo et al.^(^ ^63^ ^)^
LGR4	Stem cells of the apical papilla (SCAPs)	Mouse	Promotes odontoblast differentiation	Zhou et al.^(^ ^80^ ^)^
LGR6	BMSCs	Mouse	Upregulated during early in vitro osteogenic differentiation, with expression lost in mature osteoblasts; inhibition of LGR6 promotes BMSC osteogenic differentiation; transplant of BMSCs with a knockdown of LGR6 enhances skeletal repair in a rat fracture model	Khedgikar and Lehoczky^(^ ^65^ ^)^; Cui et al.^(^ ^75^ ^)^
LGR6	MC3T3‐E1 cells (preosteoblast cell line)	Mouse	Upregulated during early in vitro osteogenic differentiation, promotes osteogenesis via stabilization of β‐catenin; may act downstream of the BMP pathway to upregulate osteogenic genes	Liu et al.^(^ ^66^ ^)^; Zhou et al.^(^ ^67^ ^)^

## LGRs in Skeletal Development

Although R‐spondins have been reported to be major determinants of limb and craniofacial development,^(^ ^44^, ^45^, ^46^, ^47^ ^)^ expression and function of LGRs in developmental processes of bone has remained largely unclear. LGR4 is expressed in the bone collar and primary spongiosa of the developing femur diaphysis, as well as perichondrium, resting/proliferative/hypertrophic zones of cartilage in long bones, and the endosteal lining of the bone cavity in mice at E16.5.^(^ ^48^ ^)^ At E14.5, LGR4 and LGR5 are present in developing limb mesenchyme, whereas LGR6 is expressed in the overlying ectoderm of the apical ectodermal ridge, where bone morphogenetic protein (BMP) and Wnt signals control the outgrowth that results in limb formation.^(^ ^49^, ^50^ ^)^ Others have previously shown that LGR5 is expressed in the distal limb bud as early as E11.5, as well as in mesenchyme overlying the mandibular cleft prominence that later develops into the mandible.^(^ ^51^ ^)^

In an LGR4‐KO mouse model developed used a gene trap technique,^(^ ^52^ ^)^ Luo and colleagues report that only 40% of pups survived postnatally, with a developmental growth retardation characterized by shortening of the long bones. These LGR4‐KO embryos exhibited a significant delay in bone formation, specifically in limbs, jaws, and calvaria at E14.5, and in phalanges and skull bones at E16.5. The authors additionally describe a delay in the initiation of osteogenesis of cartilaginous templates at E14.5 between WT and LGR4‐KO mice. Together, these results demonstrate that LGR4 expression is important for endochondral ossification and skeletal development.^(^ ^48^ ^)^

In comparison with LGR4, LGR5 and LGR6 have been only sparsely explored in limb and skeletal development. Additionally, the LGR5‐ and LGR6‐KO mouse models currently in use are not traditional KOs, and instead rely on the insertion of EGFP‐ires‐CreER^T2^ knock‐in alleles to disrupt endogenous gene expression. In studies of these LGR‐null models, LGR5‐KO mice are nonviable and die within 24 hours after parturition, but present with a neonatal cleft palate‐like phenotype, suggesting LGR5 is involved in craniofacial development.^(^ ^53^ ^)^ LGR6‐KO mice appear to develop normally with no apparent or overt skeletal phenotype.^(^ ^1^, ^7^ ^)^

Expression of LGR homologs has additionally been examined in a zebrafish model during development, where both LGR4 and LGR6 expression is detected in cranial cartilages.^(^ ^54^ ^)^ These LGRs are specifically present in Meckel's cartilage of the mandible, which is normally resorbed in adults, yet has the ability to undergo ossification under BMP signaling pathway cues.^(^ ^55^, ^56^, ^57^ ^)^

Interestingly, recent evidence points to an LGR‐independent mechanism for R‐spondins in limb development, where a triple transgenic LGR4/5/6 KO mouse model did not recapitulate the striking limb phenotype of RSPO2^−/−^ embryos.^(^ ^49^ ^)^ Despite the possibility that RSPO‐mediated limb formation may operate through an alternative mechanism,^(^ ^58^ ^)^ it is clear that LGRs themselves play a role in skeletal development.

## LGRs in Skeletal Remodeling

There is strong evidence that LGRs are implicated in bone maintenance and homeostasis. Luo and colleagues showed that mature bone formation kinetics, as well as BMD, is negatively affected in a KO mouse model of LGR4.^(^ ^48^ ^)^ This same study determined that LGR4 has the ability to positively regulate expression of osteoblast‐specific maturation genes, including *Ocn*, bone sialoprotein (*Bsp*), and type I collagen (*Col1a1*).^(^ ^48^ ^)^ In adult bones, LGR4 expression is limited to the endosteum and periosteum, which are the tissue layers that are known to harbor osteochondral progenitor cells, as well as precursor cells within the tendon enthesis.^(^ ^59^, ^60^ ^)^ Combined, these findings implicate LGR4+ cells in osteoblast‐lineage differentiation during homeostatic bone remodeling. Expression of LGR5 and LGR6 in fully developed bones during bone remodeling remains unclear, but recent GWAS studies have shown that the LGR6 gene is a rare variant associated with risk of postmenopausal osteoporosis.^(^ ^61^ ^)^

LGRs may also mediate osteoclast activity and function during skeletal remodeling, as LGR4 is a newly described receptor for RANKL, a major regulator of osteoclast differentiation. Binding of LGR4 to RANKL has been shown to downregulate RANK in HEK293T cells, abrogating the RANK/RANKL interaction that controls differentiation of mature osteoclasts.^(^ ^62^, ^63^ ^)^ These findings corroborate with evidence that, in humans, rare nonsense mutations of the LGR4 gene are linked with osteoporosis.^(^ ^61^, ^64^ ^)^

## LGRs in Osteogenesis and Skeletal Regeneration

LGR4 and LGR5 have both been found to be expressed in osteoblast precursors and are implicated in in vitro osteogenesis. LGR6 expression levels correlate with the transcription factor *Sp7* in the early phases of murine bone marrow‐derived stromal cell (BMSC) osteogenic differentiation, presenting LGR6 as a novel candidate marker of osteoblastic progenitors.^(^ ^65^ ^)^ LGR6 expression is subsequently lost as cells differentiate into mature osteoblasts expressing high levels of the later osteogenic marker, *Ocn*. Other in vitro studies demonstrate that LGR6 expression is significantly upregulated during early osteogenic differentiation in MC3T3‐E1 cells,^(^ ^66^ ^)^ and that forced overexpression of LGR6 in this preosteoblast cell line using lentiviruses promotes osteogenesis in a β‐catenin dependent manner.^(^ ^66^ ^)^ A recent study has demonstrated that LGR6 acts to potentiate Wnt/β‐catenin signaling in MC3T3‐E1 cells downstream of BMP signaling, leading to transcription of osteogenic genes.^(^ ^67^ ^)^

LGR4 is expressed within BMSCs undergoing osteogenic differentiation; however, the expression levels of LGR4 do not appear to be correlated with osteogenic genes or in vitro osteoblastic differentiation of BMSCs.^(^ ^65^ ^)^ Although LGR4 may not serve as a marker for osteoblast precursors in the same manner as LGR6, others have demonstrated a necessary role for LGR4 in osteogenic differentiation in vitro. RSPO2 promotes osteoblast formation and activates canonical Wnt signaling in a MC3T3‐E1 preosteoblast cell line, an effect that is abrogated upon siRNA‐induced knockdown of LGR4^(^ ^68^ ^)^; RSPO2 has also been shown to induce bone formation in vivo, although studies to determine receptor activation during this process are needed.^(^ ^69^ ^)^ Further, bone marrow‐derived mesenchymal stem cells (MSCs) isolated from LGR4‐KO mice show reduced osteoblastic differentiation.^(^ ^62^ ^)^ Zhang and colleagues also report a LGR4‐mediated positive effect on osteogenic differentiation in human adipose‐derived stem cells, induced by RSPO3.^(^ ^70^ ^)^ Interestingly, this group found that the effect of RSPO3‐LGR4 on differentiation toward the osteoblast lineage is mediated through ERK/FGF signaling, rather than through the Wnt pathway.^(^ ^70^ ^)^ Addition of BMP2 to MC3T3‐E1 cells or calvarial osteoblasts upregulates LGR4 gene expression, indicating that LGR4 is a possible downstream transcriptional target of the BMP pathway during osteogenic differentiation. Functionally, a specific knockdown of LGR4 in MC3T3‐E1 cells reduces BMP2‐induced alkaline phosphatase activity.^(^ ^71^ ^)^ Combined, these results suggest that LGR4 has an overall positive effect on osteogenic differentiation, paralleling in vivo studies.^(^ ^48^ ^)^

LGR expression may also play a role in skeletal regeneration and fracture healing, processes that are dependent on various sources of osteoprogenitors.^(^ ^60^, ^72^, ^73^ ^)^ In vivo, LGR6 marks a required MSC population in a murine blastema model, where lineage‐tracing studies revealed that LGR6+ cells from the nailbed differentiate into osteoblasts of the regenerated digit tip.^(^ ^7^ ^)^ This group describes impairment of murine digit tip regeneration in LGR6‐null mice, implicating LGR6 in regeneration using a murine blastema model. In these studies, LGR5 marks a unique population of mesenchymal cells that do not contribute to regeneration during digit tip regeneration.^(^ ^7^ ^)^ This pattern, where LGR4 and LGR5 mark distinct cell populations from LGR6, is a recurrent motif in Wnt‐driven progenitor cell niches.^(^ ^1^, ^6^, ^7^, ^74^ ^)^ Although there have been no conclusive studies on LGR‐mediated fracture healing, injection of BMSCs with a knockdown of LGR6 using shRNA at the injury site of femoral fractures in rats has been reported to inhibit skeletal repair^(^ ^75^ ^)^; however, the mechanism for this remains unclear. In the context of repair these results are difficult to interpret, as Wnt signaling has a complex role in fracture repair, where it can either promote or inhibit differentiation of osteoprogenitors depending on the maturation phase of the cell when Wnt signaling becomes active.^(^ ^39^, ^40^ ^)^ This prominent focus on LGR6 in studies of osteogenesis as opposed to other LGR family members is not surprising, as LGR6 has been specifically implicated in regenerative processes of other tissues.^(^ ^1^, ^6^, ^7^, ^76^, ^77^ ^)^

## LGRs in Dental Cells and Tissues

LGRs and R‐spondins are known to be expressed in developing mineralized tissues of the oral cavity. In the developing murine molar, LGR4, LGR5, and LGR6 exhibit dynamic spatiotemporal expression, as shown via in situ hybridization.^(^ ^78^ ^)^ Specifically, LGR4 and LGR5 are most strongly detected in the dental lamina and tooth bud epithelium, whereas later stages of tooth development (eg, bell and cap stages) feature prominent LGR6 expression in the underlying mesenchyme and dental papilla. Recently, it was shown that LGR4 is required for proper sequential molar development, as keratinocyte‐specific loss of LGR4 results in failed molar formation, with a developmental defect likely caused by abnormal differentiation of the dental epithelium; this is a consequence of reduced Wnt/β‐catenin signaling during the developmental process, shown via downregulation of LEF1.^(^ ^79^ ^)^ In parallel, siRNA knockdown of LGR4 in stem cells of the apical papilla (SCAPs) isolated from developing tooth roots was shown to inhibit the process of in vitro odontogenic differentiation, and this was correlated with decreased amounts of stabilized β‐catenin.^(^ ^80^ ^)^

LGRs are also expressed in compartment‐specific locations in incisors. In the labial cervical loop, LGR4 and LGR5 label distinct areas of the epithelial portion of the enamel organ, whereas LGR6 is confined to the mesenchyme and additionally labels enamel‐producing ameloblasts. These findings suggest that LGR+ cells may give rise to various tissues of the tooth, including dentin, cementum, enamel, and the supporting periodontal ligament (PDL). LGR5 is expressed in the proximal end of murine incisors (E13.5),^(^ ^78^ ^)^ which contain a highly established stem cell niche,^(^ ^78^, ^81^, ^82^ ^)^ with an expression pattern that correlates with where transit‐amplifying cells of the tooth are found.^(^ ^83^ ^)^ However, these LGR5+ epithelial stem cells of the murine incisor do not appear to regulate Wnt/β‐catenin signaling.^(^ ^84^ ^)^ Though studies on LGR family members in fully developed teeth are lacking, LGR4 is strongly expressed in ameloblasts and odontoblasts of adult mice.^(^ ^59^ ^)^

LGR4 and LGR5 have been shown to be expressed in the PDL, a mechanoresponsive connective tissue that plays a major role in tooth movement and skeletal remodeling in alveolar bone of the tooth socket.^(^ ^85^, ^86^, ^87^, ^88^ ^)^ Expression of LGR4 in particular was reported in an immature human PDL cell line (2 to 14 cells).^(^ ^89^ ^)^ Under orthodontic strain in a rat model, LGR5 expression in vivo localizes with Runx2+ cells, specifically in the periapical region of the PDL during tooth movement and alveolar bone remodeling under the influence of these cyclic strain techniques.^(^ ^90^ ^)^ Further, LGR5+ adult stem cells have been isolated directly from the human PDL, and gene expression analyses on this population suggest that LGRs may mark PDL‐derived epithelial stem cells.^(^ ^91^ ^)^

LGRs may also mediate osteoregenerative processes within the PDL. The PDL harbors a progenitor population with differentiation potential encompassing cementoblasts, odontoblasts, osteoblasts, and fibroblasts,^(^ ^85^, ^88^ ^)^ and is partially responsible for regeneration of a subset of dental tissues following injury.^(^ ^92^, ^93^ ^)^ Addition of RSPO2 to an undifferentiated human PDL cell line expressing LGR4 induces more robust osteogenesis, although it is still unproven as this is mediated through a RSPO–LGR interaction.^(^ ^89^ ^)^ An LGR‐mediated mechanism may be accelerating osteogenic differentiation of tooth‐associated progenitor cells, similar to in vitro results seen in studies with bone marrow‐derived cells and established cell lines.^(^ ^65^, ^66^ ^)^

## Discussion

There is an emerging role for LGRs in the context of osteogenesis and skeletal regeneration. Within the field of bone biology, identifying stem cell markers of the skeleton, as well as the signaling pathways that define their behavior and function, remains an important and increasingly appreciated area of study.

A comprehensive signaling mechanism that regulates progenitor cells after injury remains undefined, but it is well‐known that the Wnt pathway is highly influential in both skeletal development and the fracture‐healing process.^(^ ^30^ ^)^ Although there are a few studies demonstrating LGR‐mediated Wnt signaling in the context of osteogenesis and bone‐associated cells, there are still unknown mechanisms and ligand interactions for multiple LGR‐associated phenotypes seen in bone. This emphasizes an important secondary role for LGRs in stem cell biology beyond their role as modulators of Wnt signaling.

LGRs are associated with human bone diseases and phenotypes. Considering the soluble nature of their known R‐spondin ligands,^(^ ^14^ ^)^ LGRs are potential therapeutic targets for a variety of bone diseases including osteoporosis, as well as defects in skeletal repair. Manipulation of LGR+ cells in regenerative medicine applications within other fields has been demonstrated: in vitro expansion of Lgr + cells and organoids via RSPO stimulation has been shown in liver and intestinal cells,^(^ ^94^, ^95^, ^96^ ^)^ and direct treatment with LGR+ cells has been attempted in dermatological wounds, where these cells appear to promote vascularization and epithelialization during the healing process.^(^ ^76^ ^)^

With multiple studies demonstrating how Wnt treatment can be harnessed to modulate bone formation and improve bone healing, it is likely that the use of R‐spondins as agonists for LGR‐mediated Wnt signaling and induction of LGR+ cells holds strong therapeutic promise.

## Disclosures

The authors have nothing to disclose.
